# Falciparum malaria in European tourists to the Dominican Republic.

**DOI:** 10.3201/eid0605.000515

**Published:** 2000

**Authors:** T Jelinek, M Grobusch, G Harms-Zwingenberger, H Kollaritsch, J Richter, B Zieger

**Affiliations:** Ludwig-Maximilians-University, Munich, Germany. jelinek@lrz.uni-muenchen.de

## Abstract

Thirteen cases of falciparum malaria acquired by Europeans in the Dominican Republic occurred from June 1999 to February 2000. The cases were identified by the European Network on Imported Infectious Disease Surveillance (TropNetEurop).


### Falciparum Malaria in European Tourists to the Dominican Republic

**Tomas Jelinek,* Manuel Corachan,† Martin Grobusch,‡ Gundel Harms-Zwingenberger,§ Herwig Kollaritsch,¶ Joachim Richter,# and Bernd Zieger** for TropNetEurop††**
*Ludwig-Maximilians-University, Munich, Germany; †Sección de Medicina Tropical, Hospital Clinic, Barcelona, Spain; ‡Medizinische Klinik mit Schwerpunkt Infektiologie, Virchow-Klinikum, Berlin, Germany; §Institut für Tropenmedizin, Berlin, Germany; ¶University of Vienna, Austria;# Heinrich-Heine-University, Düsseldorf, Germany; **Institut für Tropenmedizin, Städtische Kliniken Dresden-Friedrichstadt, Dresden, Germany; ††European Network on Imported Infectious Disease Surveillance

Thirteen cases of falciparum malaria acquired by Europeans in the Dominican Republic occurred from June 1999 to February 2000. The cases were identified by the European Network on Imported Infectious Disease Surveillance (TropNetEurop).

Malaria, falciparum malaria in particular, represents a serious health hazard to travelers to disease-endemic areas. As international air travel to tropical destinations has become more popular, imported cases have also increased in countries where malaria is not endemic (1-3). The importance of appropriate drug prophylaxis has been stressed repeatedly (4).

Like most countries in the Caribbean, large parts of the Dominican Republic are considered low risk for falciparum malaria (5). In general, only border regions to Haiti and provinces in the northwest have been associated with endemicity. This pattern has been reversed recently: starting with an index patient in June 1999, 12 additional European patients acquired falciparum malaria in the Dominican Republic from November 1999 through February 2000. The cases were identified and reported within TropNetEurop, a sentinel surveillance network of clinical sites throughout Europe whose goal is to monitor imported infectious diseases. The network has a reporting system at sentinel clinics throughout Europe, known for its speed of reporting (usually within few days of diagnosis) and for members' sites that serve as regional referral centers.

Address for correspondence: Tomas Jelinek, University of Munich, Department of Infectious Diseases and Tropical Medicine, Leopoldstr. 5, 80802 Munich, Germany; fax: (x49)-89-336112; e-mail: jelinek@lrz.uni-muenchen.de.

All but three patients (two Spanish, one Austrian) were Germans (Table). All had traveled to Punta Cana, a town in the eastern tip of the Dominican Republic, or to nearby beach resorts. Excursions were made only to the nearest town, Higüey, which was not considered malarious. The patients were not required to and did not receive malaria chemoprophylaxis for this journey and did not practice exposure precautions. Within 1-2 weeks after their return, patients visited general practitioners or emergency rooms, reported fever, and were hospitalized after diagnoses of falciparum malaria were established by blood films. For all patients, the clinical course was uneventful, and drug treatment was successful (Table).

The clustering of cases during a comparatively short time suggests a change in the epidemiologic situation in the Dominican Republic and may herald future outbreaks among tourists. According to information from the Dominican Republic, malaria cases increased in 1999 after Hurricane George: as of November 20, 1999, 3,003 cases had been reported, compared to 2,000 cases for all of 1998. In the eastern part of the country, an outbreak of falciparum malaria among the local population was noted and traced back to Haitians working in construction. With anopheline vectors and abundant breeding sites in that area, transmission of falciparum malaria is easy (6). Current recommendations for visitors

Table. Patients with falciparum malaria from the Dominican Republic

| No. | Sex[a] | Age | Nationality | Month/year presentation | Journey | Therapy |
|---|---|---|---|---|---|---|
| 1 | F | 26 | German | 06/99 | | |
| 2 | M | 28 | German | 11/99 | 14 days in Punta Cana (honeymoon with #3) | Mefloquine |
| 3 | F | 28 | German | 11/99 | 14 days in Punta Cana (honeymoon with #2) | Mefloquine |
| 4 | F | 34 | German | 11/99 | 7 days in Punta Cana | Mefloquine |
| 5 | F | 28 | Spanish | 11/99 | 7 days in Punta Cana | Chloroquine |
| 6 | F | 45 | German | 11/99 | 14 days in Punta Cana | Atovaquone/ proguanil |
| 7 | M | 27 | German | 11/99 | Flight assistant, overnight stays in Puerto Plata (October) and Punta Cana (November) | Quinine |
| 8 | F | 30 | German | 11/99 | 10 days in Punta Cana | Mefloquine |
| 9 | F | 47 | Austrian | 12/99 | 14 days in Punta Cana | Quinine |
| 10 | F | 28 | Spanish | 12/99 | 6 days in Punta Cana | Chloroquine |
| 11 | F | 30 | German | 12/99 | 10 days in Punta Cana | Mefloquine |
| 12 | F | 31 | German | 12/99 | 16 days in Punta Cana | Chloroquine |
| 13 | M | 24 | German | 02/00 | 10 days in Punta Cana | Quinine |

[a]F = female; M = male.

to the Dominican Republic should include an antimalaria strategy and strict adherence to personal protection measures against mosquito bites.

For 1998, official statistics from the World Tourism Organization put the number of visitors from Germany to the Dominican Republic at 366,599 (7). Corresponding numbers from Austria and Spain are 30,017 and 110,782, respectively. If these numbers were used as the basis for a crude denominator, the annual incidence of falciparum malaria in tourists to the east coast of the Dominican Republic would be 2.73/100,000 for German tourists and 3.3/ and 1.8/100,000 for Austrian and Spanish tourists, respectively. No reports were received of infections among tourists from other nations, including the United States, Canada, and the United Kingdom. This may reflect use of different malaria chemoprophylaxis or exposure prophylaxis for travel to the Dominican Republic.

This report demonstrates the effectiveness and importance of sentinel surveillance methods for monitoring imported infectious diseases in Europe. Discussion of the index case among the member sites of TropNetEurop increased awareness within the network and led to the other reports within days of initial diagnosis. The malaria patients might otherwise have gone unnoticed since they were seen at different hospitals throughout Europe.

TropNetEurop receives financial support from Dr. Democh Maurmeier Stiftung and Förderprogramm für Forschung und Lehre der Medizinischen Fakultät, Ludwig-Maximilians-University, Munich, Germany.

Dr. Jelinek is head of the Research Group for Imported Diseases at the Department of Infectious Diseases and Tropical Medicine, University of Munich. His research interests include molecular mechanisms of drug resistance in falciparum malaria, mechanisms of immunity to malaria, and the epidemiology of imported infectious diseases.

### References

1. Kollaritsch H, Wiedermann G. Compliance of Austrian tourists with prophylactic measures. Eur J Epidemiol 1992;8:243-51.
2. Legors F, Danis M. Surveillance of malaria in European Union countries. Eurosurveillance 1998;3:45-7.
3. Muentener P, Schlagenhauf P, Steffen R. Imported malaria (1985-95): trends and perspectives. Bull World Health Organ 1999;77:560-6.
4. Steffen R, Fuchs E, Schildknecht J, Naef U, Funk M, Schlagenhauf P, et al. Mefloquine compared with other malaria chemoprophylactic regimens in tourists visiting East Africa. Lancet 1993;341:1299-303.
5. Malaria in the Americas, 1996. Epidemiol Bull 1997;18:1-8.
6. Castellanos P. Malaria, imported—Europe ex Dominican Rep (03): ProMED Archives [mail post] 1999 17 Dec; [1 screen]. Available from: URL: http://osi.oracle.com:8080/promed/promed.home
7. Yearbook of tourism statistics. Madrid: World Tourism Organization; 1999.

## References

[PDF_00143] Kollaritsch H., Wiedermann G. (1992). Compliance of Austrian tourists with prophylactic measures.. Eur J Epidemiol.

[PDF_00146] Legros F., Danis M. (1998). Surveillance of malaria in European Union countries.. Euro Surveill.

[PDF_00148] Muentener P., Schlagenhauf P., Steffen R. (1999). Imported malaria (1985-95): trends and perspectives.. Bull World Health Organ.

[PDF_00151] Steffen R., Fuchs E., Schildknecht J., Naef U., Funk M., Schlagenhauf P., Phillips-Howard P., Nevill C., Stürchler D. (1993). Mefloquine compared with other malaria chemoprophylactic regimens in tourists visiting east Africa.. Lancet.

